# VERTICAL GASTRECTOMY VS. EXTENDED VERTICAL GASTRECTOMY: WHAT IS THE IMPACT ON GASTROESOPHAGEAL REFLUX DISEASE IN OBESE RATS?

**DOI:** 10.1590/0102-672020190001e1513

**Published:** 2020-08-24

**Authors:** José Aparecido VALADÃO, Plinio da Cunha LEAL, Eduardo José Silva Gomes de OLIVEIRA, Orlando Jorge Martins TORRES, Luis Eduardo Veras PINTO, Danilo Dallago De MARCHI, Ozimo Pereira GAMA-FILHO, Marco Aurelio SANTO, Paulo Afonso Nunes NASSIF

**Affiliations:** 1 Postgraduate Program in Principles of Surgery, Evangelic Mackenzie Faculty of Paraná/Medical Research Institute, Curitiba, PR, Brazil; 2 Federal University of Maranhão, São Luis, MA, Brazil; 3 Presidente Dutra University Hospital, São Luis, MA, Brazil; 4 Gastromed - Zilberstein Institute, São Paulo, SP, Brazil; 5 Department of Gastroenterology, Faculty of Medicine, University of São Paulo, São Paulo, SP, Brazil

**Keywords:** Gastrectomy, Gastroesophageal reflux, Rats, Bariatric surgery, Gastrectomia, Refluxo gastroesofágico, Ratos, Cirurgia bariátrica

## Abstract

**Background:**

Extended vertical gastrectomy is a variation of the vertical gastrectomy technique requiring studies to elucidate safety in relation to gastroesophageal reflux.

**Aim:**

To analyze comparatively vertical gastrectomy (VG) and extended vertical gastrectomy (EVG) in rats with obesity induced by cafeteria diet in relation to the presence of reflux esophagitis, weight loss and macroscopic changes related to the procedures.

**Methods:**

Thirty Wistar rats were randomized into three groups, and after the obesity induction period by means of a 28-day cafeteria diet, underwent a simulated surgery (CG), VG and VGA. The animals were followed up for 28 days in the post-operative period, and after euthanasia, the reflux esophagitis evaluation was histopathologically performed. Weight and macroscopy were the other variables; weight was measured weekly and the macroscopic evaluation was performed during euthanasia.

**Results:**

All animals presented some degree of inflammation and the presence of at least one inflammation criterion; however, there was no statistically significant difference in the analysis among the groups. In relation to weight loss, the animals in CG showed a gradual increase during the whole experiment, evolving to super-obesity at the end of the study, while the ones with VG and EVG had weight regain after the first post-operative period; however, a less marked regain compared to CG, both for VG and EVG.

**Conclusion:**

There is no difference in relation to reflux esophagitis VG and EVG, as well as macroscopic alterations, and both techniques have the ability to control the evolution of weight during postoperative period in relation to CG.

## RESUMO

**Racional:**

A gastrectomia vertical ampliada é uma variação da técnica da gastrectomia vertical, necessitando de estudos a fim de elucidar a segurança em relação ao refluxo gastroesofágico.

**Objetivo:**

Analisar comparativamente gastrectomia vertical (GV) e gastrectomia vertical ampliada (GVA) em ratos com obesidade induzida por dieta cafeteria em relação à presença de esofagite de refluxo, perda de peso e alterações macroscópicas relacionadas aos procedimentos.

**Método:**

Trinta ratos Wistar foram randomizados em três grupos, e após período de indução de obesidade por meio de dieta cafeteria de 28 dias, foram submetidos a operação simulada (grupo controle GC), gastrectomia vertical (grupo GV) e gastrectomia vertical ampliada (grupo GVA). Os animais foram acompanhados por 28 dias no pós-operatório e, após a eutanásia, foi realizada a pesquisa de esofagite de refluxo através de avaliação histopatológica. Peso e avaliação macroscópica foram as outras variáveis de estudo, sendo o peso aferido semanalmente e a avaliação macroscópica no momento da eutanásia.

**Resultados:**

Todos os animais apresentaram algum grau de inflamação e a presença de ao menos um critério de inflamação, porém, não houve diferença estatisticamente significante na análise entre os grupos. Em relação à perda de peso, os animais do GC apresentaram aumento gradativo durante todo experimento evoluindo para super-obesidade ao término do estudo, enquanto os dos grupos GV e GVA tiveram reganho de peso após a primeira semana do pós-operatório, porém, reganho menos acentuado se comparável ao GC, tanto para GV quanto para GVA.

**Conclusões:**

Não há diferença em relação à esofagite de refluxo entre GV e GVA, bem como em relação às alterações macroscópicas. Ambas as técnicas têm capacidade de controlar a evolução do peso no pós-operatório em relação ao grupo controle.

## INTRODUCTION

Vertical gastrectomy (VG) has not yet had its surgical technique fully standardized. The procedure consists of removing an extensive part of the large curvature of the stomach, part of the body and the entire gastric fundus, making a reservoir of smaller volume and tubular shape^29^. However, there is still no standardization as to the extent of resection of the antropyloric region, which is maintained in a greater or lesser proportion, and the final volume of the gastric reservoir^15^.

The extended vertical gastrectomy (EVG) is a technical variation of the VG proposed by Nassif et al^18^. In this technique, the first staples are parallel to the largest axis of the pylorus, narrowing the antropyloric region. The dissection is guided by a 32F gauge probe, allowing the production of a tubular stomach, with a standardized volume, thus avoiding stenosis. The dissection ends by removing the largest portion of the body and the entire gastric fundus. Therefore, the final aspect of the organ is thinner and more uniform.

Gastroesophageal reflux disease (GERD) is a possible complication of VG, as it seems to increase the incidence of GERD and/or worsen pre-existing reflux^14^. Studies using 24-hour impedance-pH-monitoring to assess the presence of gastroesophageal reflux in patients undergoing VG concluded that 50% of those ones studied started to have GERD and that it worsened in 80% of those who already had the disease^8^. The presence of GERD was pointed out as a contraindication for VG by 57% of specialists during the International Sleeve Gastrectomy Expert Panel Consensus Statement in 2012^24^. However, the subject remains controversial, and there is still no consensus in the literature on the subject^1,3,5,20,21,31,32, 35,36^.

In this context, experimental studies aiming at elucidating the relation between VG and GERD are of great importance, since the aspects that address this surgical treatment for obesity and GERD are divergent in the literature and should be further investigated.

Therefore, the aim of this research was to compare VG and EVG techniques in a single experimental study regarding to GERD, as they are surgical treatments for obesity.

## METHOD

The research was carried out after the approval by the Ethics Committee on the Use of Animals of the Federal University of Maranhão (Protocol 23115.012273/2015-08; CEUA registration: 35/15).

### Animals and experimentation environment

The sample consisted of 30 adult male rats of the species *Ratus novergicus albinus*, Wistar, with an average weight of 250 g from the Vivarium of the Federal University of Maranhão. During the whole experiment, they were under noise and temperature control (23±1°C), with a 12-hour light/dark cycle being maintained and hygiene conditions guaranteed and changing out whenever necessary the Xilana^*®*^ used as a lining for the cages.

### Food and water

The animals went through a seven-day period of adaptation, during which they received standard Purilab^®^ food and filtered water ad libitum. Then, the cafeteria-type hyper caloric diet was introduced, which was maintained from the beginning of the fattening phase up to the moment of the last experimental stage with the euthanasia of the animals, except in the 8 h preceding the surgical procedures in which they remained fasting and in the immediate postoperative period (first 24 h), in which they did not receive a solid diet, only a liquid one.

### Experimental design

**FIGURE 1:**
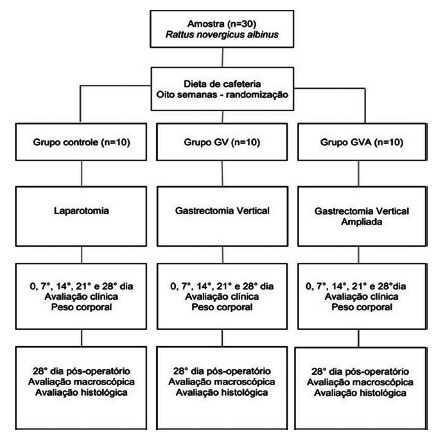
Study Design

All animals received surgical treatment from the same researchers and in the same study period. The obesity induction period was 28 days, and the postoperative follow-up was also 28 days when they were euthanized (Figure 1).

The rats were randomized and subdivided as per the surgical technique to which they would be submitted to, creating three groups of 10 animals each: control group (CG), vertical gastrectomy group (VG) and extended vertical gastrectomy group (EVG). In the animals of CG, only simulated operation was performed via bi-digital manipulation of the stomach; in VG a vertical gastrectomy and in the EVG group an extended vertical gastrectomy were done.

The experiment was carried out in the experimental surgical center of the Experimental Surgery Laboratory of the Federal University of Maranhão, São Luis, MA, Brazil. After the adaptation period, the animals were weighed and randomly distributed to compose the three study groups as per the surgical technique to be employed and the period of obesity induction commenced by means of a high-calorie cafeteria-type diet.

### Induction of obesity via hypercaloric cafeteria diet

In order to produce obesity, the cafeteria diet was used, so named because it contains hyper energetic foods, consisting of a solid part associated with the standard ration composing a high-calorie diet; it is produced by hand, by mixing crushed foods, containing 500 g of bacon, 1 kg of roasted peanuts, 1 kg of cornstarch biscuit, 500 g of milk chocolate and a liquid part of filtered water and Guaraná Jesus^®^ soft drink, hyper caloric liquid. All foods, both the standard diet and the cafeteria diet, were offered ad libitum throughout the experiment.

This hypercaloric diet protocol was analyzed by the Physiology Laboratory of the Federal University of Maranhão, and it was determined that it had 506.2 kcal/100 g, and a nutritional value consisting of 35.3% carbohydrates, 34.5% lipids and 15.4% protein. According to the manufacturer, the Guaraná Jesus^®^ soft drink had 53.1 kcal/100 ml and contained 12 g/100 ml of carbohydrates.

The animals’ weight was measured weekly throughout the experiment, being considered obese rats the ones that increased their weight by 30% prior to the commencement of the high-calorie diet.

### Surgical procedure and sample handling

The animals were fasted for 8 h before the surgical procedure was performed under anesthesia with a combination of 10% ketamine hydrochloride at a dose of 100 mg/kg and 2% xylazine hydrochloride at a dose of 10 mg/kg, applied intraperitoneally, using a syringe and insulin needle, after the animal was contained manually. Then, they were placed in the dorsal decubitus position on a 15x15 cm wooden plank and fixed with surgical adhesive tape, epilated in the incision region, antisepsis was performed with polyvinylpilorridone iodine in 10% alcohol solution and a sterile fenestrated surgical drape was placed over the animal.

Access to the abdominal cavity was laparotomic, by means of dieresis until the opening of the peritoneal cavity, approximately 5 cm from the xiphoid appendix through the midline of the abdomen, using a disposable cold scalpel with a #15 blade.

In the 10 animals in the CG, after accessing the abdominal cavity, orogastric cannulation was performed using a nelaton probe #8, to identify the stomach and bidigital manipulation of the ventral and dorsal walls of the gastric body. Then, the abdominal wall was closed by using continuous suture with 4.0 polyglactin thread and the skin was closed by using continuous intradermal suture with the same thread.

In the 10 animals of the VG group, also after orogastric cannulation using it to identify the stomach, a proximal point located on the gastric juxta-esophageal fundus and another distal located 15 mm from the pylorus was marked for anatomical reference. The gastric excision plane was demarcated with a Crile hemostatic forceps, followed by the dieresis and excision of the gastric fundus, part of the body and antrum in the great curvature of the stomach, with subsequent closure of the dieresis line with a continuous extramucosal suture with 5.0 polyglactin thread. Then, the abdominal wall was closed in the same way as described above and the skin was closed by continuous intradermal suture with 4.0 polyglactin thread.

In the 10 animals in the EVG group, after accessing the abdominal cavity, the same procedure was performed to identify the stomach. As an anatomical reference, a proximal point located on the juxtaesophageal gastric fundus and another distal point located 5 mm from the pylorus was used. The gastric excision plane was demarcated by two hemostatic clamps, followed by the dieresis and excision of the gastric fundus, part of the body and antrum in the great curvature of the stomach, with subsequent closure of the dieresis line with extramucosal continuous suture with a 5.0 polyglactin thread. Then, the abdominal wall and skin were closed as described in the VG.

In the immediate postoperative period (first 24 h), the animals were fasted to solids, with access to water with glucose (two 50% ampoules in 500 ml of water) ad libitum. From the second day onwards, the entire diet was reintroduced, that is, standard ration and filtered water and solid and liquid cafeteria diet. Postoperative analgesia was performed in the first 72 h after the procedure, using paracetamol orally in the dose of one drop (10 mg) for every 25 ml of water.

Euthanasia was performed using an overdose of anesthetic, applying four times the dose used to perform anesthesia. Immediately after euthanasia and the confirmation of death, an exploratory laparotomy was performed with the assessment over secretions and adhesions within the abdominal cavity, following the parameters of Nair et al^16^. After the macroscopic assessment and classification of adhesions, the stomach was removed together with the distal third of the esophagus in a single piece to perform the anatomopathological study and to assess the presence of distal esophagitis as a manner to identify the presence of gastroesophageal reflux. 

The presence of esophagitis was investigated through the inflammation found during the analysis, being classified as mild, moderate and severe, thus receiving the score grade 1 for mild, 2 for moderate and 3 for severe. Esophagitis was also investigated using the histopathological criteria for inflammation: papilla elongation, hyperkeratosis, hypergranulose, mucosal muscle atrophy, exocytosis, vascular congestion and neovascularization, following the model proposed by Gaia et al^7^. 

### Statistical analysis

The data were assessed using the NCSS 11 software (2016). To assess the effect of the three groups and the weeks in relation to the weight-dependent variable, the Shapiro-Wilk test was initially performed; as all measures showed normal distribution (p> 0.05), the analysis of variance test (ANOVA) and Tukey’s post-hoc test were applied to compare groups two by two and to compare weeks two by two. The assessment of the effect of the groups on ordinal dependent variables, such as the classification of NAIR and inflammation, was performed using the Kruskall-Wallis non-parametric test. Subsequently, categorical variables (papilla elongation, hyperkeratosis, hypergranulose, mucosal muscle atrophy, exocytosis, vascular congestion and neovascularization) were assessed using the non-parametric chi-square test of independence (χ^2^). A value of p<0.05 was considered statistically significant.

## RESULTS

### Change in body weight in the period of obesity induction

**FIGURE 2:**
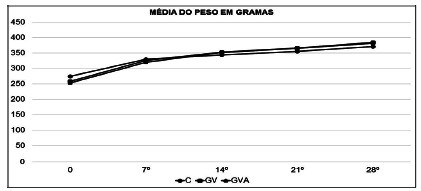
Evolution of body weight during the period of obesity induction in the control (CG), vertical gastrectomy (VG) and extended vertical gastrectomy (EVG) groups

The average weight of the animals prior to the start of induction was 274.7 g in the CG, 257.8 g in the VG and 253.4 in the EVG. After 28 days of feeding with the high-calorie cafeteria diet, the animals reached an average weight of 371 g (CG), 384 g (VG), 382.1 g (EVG). The weekly weight gain was equivalent in all groups, with no significant difference in the intergroup comparison (Figure 2).

### Changes in body weight in the postoperative period

The CG maintained weight gain even in the first postoperative week, maintaining a linear gain pattern throughout the follow-up period, evolving to super-obesity over the eight weeks of the experiment.

In turn, VG and EVG showed weight loss in the first postoperative week, this loss being equivalent in both groups, with no statistical difference in the comparison between VG and EVG. From the second postoperative week, both VG and EVG started to show weight regain, maintaining it until the end of the experiment, reaching the end of it with a weighted average equivalent to the moment when the operations were performed.

**FIGURE 3:**
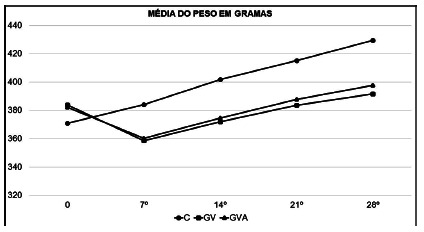
Evolution of body weight in the postoperative period - intergroup evaluation

 Thus, it was observed that in the weight evolution after the first week there was a significant difference (p=0.05) after VG and EVG, both of which differed from the CG. However, there was no significant difference (p>0.05) between VG and EVG. The weight evolution of the three groups is found in a comparative way shown in Figure 3.

### Macroscopic evaluation

All animals showed good healing with anatomical reconstitution of the skin and abdominal wall. Those in the CG had an anatomically healthy stomach. In VG and EVG, the stomach suture line showed no signs of dehiscence or local infection.

Adhesions occurred with adjacent organs in all groups. The main site was between the VG and EVG stomach suture with the liver, small intestine and abdominal wall. In the analysis by the Nair score, no significant differences were found between the groups (Table 1).

**TABLE 1:** Kruskal-Wallis test of the NAIR score

Variable	Median	IQ	P
NAIR			
Control	1	(1 - 1)	0,064
Vertical gastrectomy	1,5	(1 - 2)	
Extended vertical gastrectomy	1,5	(1 - 2)	

IQ= interquartile range

**FIGURE 4:**
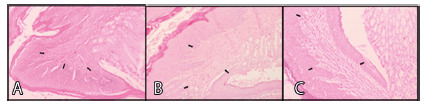
A) Mild inflammation - grade 1; B) moderate inflammation - grade 2; C) acute inflammation - grade 3 (40x, H&E)

**FIGURE 5:**
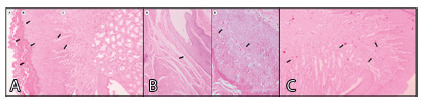
A) Hyperkeratosis (a), hypergranulose (b) and papilla elongation (c); B) mucosal muscle atrophy (a) and exocytosis (b); C) neovascularization and vascular congestion (40x, H&E)

All animals in each group had the inflammation found at the esophagogastric junction classified as mild, moderate and severe, receiving a score of 1, 2 and 3, respectively (Figures 4 and 5). The comparative statistical analysis among the three groups concluded that there was no significant difference (p>0.05) in the classification of inflammation (Table 2).

**TABLE 2:** Kruskal-Wallis test for inflammation classification

Variable	Median	IQ	P
Inflammation			
Control	2	(2 - 2)	0,201
Vertical gastrectomy	2	(2 - 2)	
Extended vertical gastrectomy	3	(2 - 3)	

IQ= interquartile range

For esophagitis research carried out using the histopathological criteria of inflammation (papilla elongation, hyperkeratosis, hypergranulosis, mucosal muscle atrophy, exocytosis, vascular congestion and neovascularization), positive and negative were assigned in relation to the presence or absence of the criteria to be evaluated (Figures 5 A, B and C). The comparative statistical analysis between the groups in relation to the criteria considered, concluded that there was a significant difference only in relation to exocytosis among the three study groups (p<0.05).

## DISCUSSION

The protocol adapted by the Experimental Surgery Laboratory of the Federal University of Maranhão was used in this experiment as a means of inducing obesity by the cafeteria diet aiming at better mimicking the habits that lead to human obesity, consisting of a solid part and a liquid part by means of soft drink^23^ ^,^ ^25^.This diet offers, in addition to a greater variety of foods, more palatable foods inducing increased food intake and, hence, body weight gain and obesity, through increased abdominal visceral fat and greater lipid accumulation in adipocytes, compared to models that the addition is done only by means of fat overload^27^. The protocol employed is considered hyper-caloric compared to the standard diet, with the energy increase in the diet being attributed, above all, to the increase in the proportion of lipids in the diet (34.5% of lipids in the cafeteria vs. 4% in the standard diet). Another aspect of great importance in this protocol is the liquid component of the diet, by means of the soft drink Guaraná Jesus^®^, which significantly increased the energy intake of the diet (53.1 kcal/100 ml).

Thus, the model employed proved to be effective in inducing obesity in the research, and after the 28-day period all animals developed obesity and were able to be qualified to the experiment, that is, there was 100% efficiency in induction.

After VG and EVG weight loss occurred only in the first postoperative week, whereas from the second week onwards they start regaining weight, reaching the end of eight weeks with a weight similar to the one on the time of the operation. A relevant fact to be underlined is the cafeteria diet was maintained throughout the experiment, allowing a large caloric intake even with the restrictive component of the operation. Another aspect of great relevance is the comparison of VG and EVG in relation to CG. The animals in this group showed linear weight gain and evolved to super-obesity compared to VG and EVG, whereas there was no difference in the analysis between VG and EVG.

Therefore, this study differs from several other studies found in the literature that showed weight loss after performing a vertical gastrectomy, both those ones performed without an intestinal stapler^10^ ^,^ ^13^ ^,^ ^30^ ^,^ ^34^ and those performed with it^22^ ^,^ ^26^. One issue that stands out is the comparison with the research carried out by Valentí et al^34^ that showed the ability to lose weight with and without maintaining a cafeteria diet in rats submitted to VG. The key point that differentiates the two studies is the presence of hyper-caloric fluid in the cafeteria diet protocol used in this research, which allows explaining the divergence of the results found. 

The results achieved here in relation to VG and EVG corroborate to those results found by Bielohuby et al^2^, in which showed that a high-calorie diet rich in carbohydrates is capable of leading to weight regain in rats submitted to VG.

Therefore, the weight regain found in all animals submitted to VG and EVG in this research may be a stimulus to the line of research, opening an opportunity to research the metabolic effects of gastrectomy, regardless of weight loss, since the metabolic role of this procedure has been the subject of several studies found in the literature^4^ ^,^ ^9^ ^,^ ^12^ ^,^ ^13^.

This study did not show any significant difference between the degrees of adherence in the analysis between the groups, which may indirectly infer the absence of major complications comparatively. These data corroborate with those found by researchers who studied the healing process and the effect of herbal medicines on gastrorrhaphy in rats^28^. 

The anatomopathological study revealed some degree of inflammation in all animals in all groups (CG, VG and EVG); however, there was no statistical difference in the comparison between groups. The esophagitis survey carried out using the histopathological criteria for inflammation also revealed the presence of these criteria in all groups, with all animals affected with some esophagitis; however, in the same way, there was no statistically significant difference in the comparison of this criterion between the groups, except in relation to exocytosis. Therefore, the results show that VG and EVG are not operations that lead to gastroesophageal reflux.

In the literature, there are no studies on reflux esophagitis after VG in rats, this research being a trailblazer in the subject, as well as in relation to EVG carried out for the first time in rats in an experimental manner. Research on rats, relating obesity and gastroesophageal reflux is also lacking in the literature. Therefore, the results achieved in this research point out to the fact that EVG does not pose greater risks for reflux esophagitis in relation to VG, and it also allows inferring the relation between obesity and GERD, since all animals, including those from the CG, presented some evidence of reflux esophagitis; however, in addition, VG and EVG controlled the evolution of weight in relation to CG; all animals were obese at the end of the experiment at the time of euthanasia.

Studies^7^ ^,^ ^11^ ^,^ ^19^ have shown by means of histopathological analysis the presence of reflux esophagitis experimentally induced in rats. Therefore, the results found in relation to the presence of reflux esophagitis in this study corroborate to those achieved in the literature.

Human studies analyzing VG and GERD still differ in relation to the results and suggest the need for further research on the subject. Nassif et al^17^, carried out a bibliographic review in order to assess the induction of gastroesophageal reflux disease in the postoperative period of VG and Roux-en-Y gastric bypass and discussed the anti-reflux barriers; the main one is the gastric fundus that is removed in the VG, making the pressure of the lower esophageal sphincter the only remaining anti-reflux barrier. Yet, these authors reinforce the hypothesis raised earlier by Nassif et al^18^ of the technical variation for EVG, explaining that instead of keeping the antrum practically intact, as it is usually done, when performing complete gastric tubulization less cubic volume could be obtained in the gastric lumen. Thus, the pressure on the lower esophageal sphincter would be lower and the amount of gastroesophageal reflux could be lower. At the end of the study, they concluded that the VG technique poses a greater compromise of the anti-reflux mechanisms predisposing the occurrence of GERD, compared to Roux-en-Y gastric bypass. 

In a systematic review, Chiu et al^5^ selected a total of 11 articles with follow-up data both before and after VG and the evolution of GERD; among them, four showed an increase in postoperative GERD and seven concluded its decrease.

Literature reviews involving VG and GERD continue to raise discussions and still do not lead to exact conclusions. Melissas et al^14^, show that VG can improve, worsen or even cause GERD, although there is consensus among the authors that VG should be contraindicated in patients with severe reflux or Barrett’s esophagus, and VG can be performed safely when there is an isolated recommendation for bariatric surgery. Crawford *et al* ^6^, when examining the different methods of anti-reflux procedures available before and after VG, point out that there is a need for further studies involving strategies to contain reflux after VG, that it must be contraindicated in patients with pre-existing severe reflux, and that the only proven method for treating reflux that is difficult to control is Roux-en-Y gastric bypass. Oor *et al* ^20^ concluded that due to the heterogeneity of studies involving the topic that lead to paradoxical results, surgeons must carefully assess the symptoms of GERD and recommend the most appropriate bariatric surgery technique.

Although one should not compare research results in different species and apply experimental results to animals in clinical practice immediately, these studies point out the need for clinical trials to improve safety in relation to the technique; the results achieved in the experiments of this research point out in favor of the applicability of VG and EVG as they do not show any difference between the techniques and the control group in relation to reflux esophagitis. However, research should be further investigated in order to better assess the presence of gastroesophageal reflux, and especially if there is reflux prior to the surgical procedure.

## CONCLUSIONS

There was no difference regarding reflux esophagitis between the VG and EVG techniques in obese rats and both were able to control weight. Also, the macroscopic changes were not different.

## References

[1] Barros F, Negrão MG, Negrão GG (2019). Weight loss comparison after sleeve and roux-en-y gastric bypass:
systematic review. Arq Bras Cir Dig.

[2] Bielohuby M, Stemmer K, Berger J (2012). Carbohydrate content of post-operative diet influences the effect
of vertical sleeve gastrectomy on body weight reduction in obese
rats. Obes Surg.

[3] Buchwald H, Estok R, Fahrbach K (2009). Weight and type 2 diabetes after bariatric surgery: systematic
review and meta-analysis. Am J Med.

[4] Chambers AP, Smith EP, Begg DP (2014). Regulation of gastric emptying rate and its role in
nutrient-induced GLP-1 secretion in rats after vertical sleeve
gastrectomy. Am J Physiol Endocrinol Metab.

[5] Chiu S, Birch DW, Shi X, Sharma AM, Karmali S (2011). Effect of sleeve gastrectomy on gastroesophageal reflux disease a
systematic review. Surg Obes Relat Dis.

[6] Crawford C, Gibbens K, Lomelin D, Krause C, Simorov A, Oleynikov D (2017). Sleeve gastrectomy and anti-reflux procedures. Surg Endosc.

[7] Gaia EV, Goldenberg A, Costa HO (2005). Experimental model of gastroesophageal reflux in
rats. Acta Cir Bras.

[8] Georgia D, Stamatina T, Maria N (2017). 24-h Multichannel Intraluminal Impedance PH-metry 1 Year After
Laparocopic Sleeve Gastrectomy an Objective Assessment of Gastroesophageal
Reflux Disease. Obes Surg.

[9] Grong E, Arbo IB, Thu OK, Kuhry E, Kulseng B, Mårvik R (2015). The effect of duodenojejunostomy and sleeve gastrectomy on type 2
diabetes mellitus and gastrin secretion in Goto-Kakizaki
rats. Surg Endosc.

[10] Kodama Y, Zhao CM, Kulseng B, Chen D (2010). Eating behavior in rats subjected to vagotomy, sleeve
gastrectomy, and duodenal switch. J Gastrointest Surg.

[11] Kranendonk S (1980). Reflux Oesophagitis: An experimental study in rats.

[12] Li F, Zhang G, Liang J, Ding X, Cheng Z, Hu S (2009). Sleeve gastrectomy provides a better control of diabetes by
decreasing ghrelin in the diabetic Goto-Kakizaki rats. J Gastrointest Surg.

[13] Lopez PP, Nicholson SE, Burkhardt GE, Johnson RA, Johnson FK (2009). Development of a sleeve gastrectomy weight loss model in obese
Zucker rats. J Surg Res.

[14] Melissas J, Braghetto I, Molina JC (2015). Gastroesophageal Reflux Disease and Sleeve
Gastrectomy. Obes Surg.

[15] Michalsky D, Dvorak P, Belacek J, Kasalicky M (2013). Radical Resection of the Pyloric Antrum and Its Effect on Gastric
Emptying After Sleeve Gastrectomy. Obesity Surgery.

[16] Nair SK, Bhat IK, Aurora AL (1974). Role of proteolytic enzyme in the prevention of postoperative
intraperitoneal adhesions. Arch Surg.

[17] Nassif FPAN, Valadão JA, Malafaia O, Torres OJM, Garcia RF, Klostemann FC (2013). Modificação técnica para a gastrectomia vertical. Arq Bras Cir Dig.

[18] Nassif PAN, Malafaia O, Ribas-Filho JM, Czeczko NG, Garcia RF, Ariede BL (2014). Vertical gastrectomy and gastric bypass in Roux-en-Y induce
postoperative gastroesophageal reflux disease. Arq Bras Cir Dig.

[19] Omura N, Kashiwagi H, Chen G, Suzuki Y, Yano F, Aoki T (1999). Establishment of surgically induced chronic acid reflux
esophagitis in rats. Scand J Gastroenterol.

[20] Oor JE, Roks DJ, Ünlü Ç, Hazebroek EJ (2016). Laparoscopic sleeve gastrectomy and gastroesophageal reflux
disease a systematic review and meta-analysis. Am J Surg.

[21] Palermo M, Serra E, Duza G (2019). N-sleeve gastrectomy: an option for obesity and
GERD. Arq Bras Cir Dig.

[22] Patrikakos P, Toutouzas KG, Perrea D (2009). A surgical rat model of sleeve gastrectomy with staple technique
long-term weight loss results. Obes Surg.

[23] Pinto DAC, Seraphim PM (2012). Cafeteria diet intake for fourteen weeks can cause obesity and
insulin resistance in Wistar rats. Rev Nutr.

[24] Rosenthal R (2012). International Sleeve Gastrectomy Expert Panel Consensus Statement
best practice guidelines based on experience of &gt;12,000
cases. Surgery for Obesity and Related Diseases.

[25] Rosini TC, Silva ASR, Moraes C (2012). Obesidade induzida por consumo de dieta: modelo em roedores para
o estudo dos distúrbios relacionados com a obesidade. Rev Ass Med Bras.

[26] Saeidi N, Nestoridi E, Kucharczyk J, Uygun MK, Yarmush ML, Stylopoulos N (2012). Sleeve gastrectomy and Roux-en-Y gastric bypass exhibit
differential effects on food preferences, nutrient absorption and energy
expenditure in obese rats. Int J Obes (Lond).

[27] Sampey BP, Vanhoose AM, Winfield HM (2011). Cafeteria diet is a robust model of human metabolic syndrome with
liver and adipose inflammation comparison to high-fat diet. Obesity (Silver Spring).

[28] Santos JO, Ribas-Filho J, Czeczko NG, L M, Dobrowlski S (2006). Avaliação do extrato de aroeira (Schinus terebinthifolius Raddi)
no processo de cicatrização de gastrorrafias em ratos. Acta Cir Bras.

[29] Shi X, Karmali S, Sharma AM, Birch DW (2010). A review of laparoscopic sleeve gastrectomy for morbid
obesity. Obes Surg.

[30] Stefater MA, Pérez-Tilve D, Chambers AP (2010). Sleeve gastrectomy induces loss of weight and fat mass in obese
rats, but does not affect leptin sensitivity. Gastroenterology.

[31] Stenard F, Iannelli A (2015). Laparoscopic sleeve gastrectomy and gastroesophageal
reflux. World J Gastroenterol.

[32] Tonatto-Filho AJ, Gallotti FM, Chedid MF, Grezzana-Filho TJM, Garcia AMSV (2019). bariatric surgery in brazilian public health system: the good,
the bad and the ugly, or a long way to go. Yellow sign. Arq Bras Cir Dig.

[33] Vale JR, Czeczko NG, Aquino JM, Nassif PAN, Henriques GS (2006). Estudo comparativo da cicatrização de gastrorrafias com e sem o
uso de extrato de Jatropha gossupiifolia L. (pião roxo) em
ratos. Acta Cir Bras.

[34] Valentí V, Martín M, Ramírez B (2011). Sleeve gastrectomy induces weight loss in diet-induced obese rats
even if high-fat feeding is continued. Obes Surg.

[35] Zeve JLM, Novais PO, O N (2012). Técnicas em cirurgia bariátrica: uma revisão da
literatura. Ciênc Saúde.

[36] Zilberstein B, Santo MA, Carvalho MH (2019). critical analysis of surgical treatment techniques of morbid
obesity. Arq Bras Cir Dig.

